# Virtual Delivery of Early Psychosis Care: Retrospective Cohort Study of Factors Associated With Initial Engagement

**DOI:** 10.2196/81313

**Published:** 2026-02-19

**Authors:** Trinity Vey, Nicole Kozloff, George Foussias, Wei Wang, Albert HC Wong, Aristotle N Voineskos, Nicole Davis-Faroque, Alexia Polillo

**Affiliations:** 1 Centre for Addiction and Mental Health, 1025 Queen Street West, Fourth Floor, Toronto, ON, M6J 1H1, Canada, 1 416 535-8501 ext 30137; 2 Temerty Faculty of Medicine, University of Toronto, Toronto, ON, Canada; 3 Department of Psychiatry, University of Toronto, Toronto, ON, Canada; 4 Dalla Lana School of Public Health, University of Toronto, Toronto, ON, Canada

**Keywords:** early psychosis intervention, psychosis, youth, virtual care, health equity, access to care, engagement

## Abstract

**Background:**

There is evidence that virtual delivery of early psychosis intervention (EPI) is well received by youth and has benefits such as reported improvements in accessibility, convenience, and comfort; however, potential barriers remain, including the digital divide and privacy concerns. Although initial engagement in EPI services is important for long-term recovery, little is known about initial engagement in the context of virtual care and the role of health equity and service use factors.

**Objective:**

This study aimed to identify factors associated with attendance at the initial EPI consultation appointment when most were being delivered virtually.

**Methods:**

This retrospective cohort study used electronic medical record data from patients aged 16 to 29 years who were referred to a large EPI program. The EPI program received 301 unique referrals that met study eligibility criteria from April to December 2020. Self-reported demographic variables were derived from the Centre for Addiction and Mental Health’s structured health equity form and included age, gender, racial and ethnic group, country of birth, and sexual orientation. Service use factors derived from clinical documentation included referral source, days to consultation, and attendance at the consultation appointment, which was the primary outcome. Comparisons were made with 2018 to 2019 data from 999 participants from the same site prior to virtual care implementation using chi-square tests for categorical variables, independent *t* tests for continuous variables, and binary logistic regression.

**Results:**

Patients had a mean age of 23.2 (SD 3.3) years, and 214 (71.1%) participants identified as male. Compared with pre–virtual care, there were significantly higher rates of inpatient referral (114/301, 37.9%) and lower rates of referral from outpatient and other providers (122/301, 40.5%) post–virtual care (*χ*^2^_2_=18.7, *P*<.001), with a small effect size and moderately narrow CI (Cramér *V*=0.120, 95% CI 0.06 to 0.17). Following univariable tests and stepwise backward selection, identifying as Black (odds ratio 0.45, 95*%* CI 0.21 to 0.97) and being referred from the emergency department or bridging clinic (odds ratio 0.24, 95*%* CI 0.08 to 0.72) were associated with decreased odds of attendance at the consultation appointment in the final adjusted model. All tests were 2-sided with an *α* level of .05.

**Conclusions:**

This study is innovative in that it examines the self-reported health equity and service use factors that may contribute to nonattendance when most EPI appointments are delivered virtually, unlike previous studies that focused solely on differences in attendance rates. Although it was during the COVID-19 pandemic and may not be representative of virtual care in real-world practice, this study suggests that virtual care may improve initial engagement in EPI services; however, barriers to care still exist for Black patients and those referred from the emergency department. A hybrid model may improve connection to EPI, though targeted approaches are needed to bridge the digital divide and ensure that structurally marginalized and high-acuity patients have equitable access to care.

## Introduction

The COVID-19 pandemic presented an urgent need to transition mental health services from in-person to virtual delivery, leading to research on feasibility and effectiveness. It also raised questions about the digital divide, referring to disparities between communities in access to technology, and the implications for structurally marginalized groups already facing barriers to accessing care [1]. Like many services, early psychosis intervention (EPI) programs rapidly shifted to virtual care during the COVID-19 pandemic, with the hope of maintaining access to and engagement in services. This was particularly crucial, given that approximately half of youth with psychosis do not access treatment and one-third disengage from EPI services early [2-5], despite the emphasis on identification and treatment early in the course of illness.

Research suggests that barriers preventing youth from accessing EPI treatment generally include stigma and a lack of knowledge about psychosis and where to seek treatment [6-8], with youth often viewing pathways to EPI care as complex [8]. Families can represent key sources of support throughout the help-seeking process and can help maintain engagement with services, particularly given the typical age of onset for psychosis [9,10]. Unfortunately, pathways to EPI treatment often begin with referrals from acute services, including emergency departments (EDs) or inpatient units [11,12], which also face barriers to ensuring timely outpatient follow-up. These include patient-level barriers, such as transportation or financial issues, and health systems–level issues, such as coordination between the ED and outpatient services, insufficient funding, and political disinterest [13,14]. Building partnerships between EPI and external services, including schools and shelters, can facilitate early detection, increase referrals from nonacute pathways, and improve access to vulnerable patients [14,15].

Emerging evidence suggests that virtual EPI care is well received by youth with psychosis. In recent cross-sectional studies, youth expressed satisfaction with the virtual delivery of EPI services and found it comparable to in-person treatment [16], highlighting the convenience, ease of use, accessibility, and comfort of virtual care [17]. Virtual care can also be conducive to a client-centered approach, bolstering youth autonomy and decision-making power over care [17]. While many youth have acknowledged the value of virtual care, some have reported feeling more isolated and disconnected from clinicians, finding it more difficult to express themselves during virtual appointments [17]. Other, more practical challenges include technological difficulties and privacy/confidentiality concerns. The extent to which the benefits of virtual care extend to improvements in initial EPI attendance is unclear.

Using service use data from EPI programs in the United States and Australia, 3 studies examined attendance rates before and after the implementation of virtual care, yielding inconsistent results. One study found an increase in missed appointments after virtual care implementation compared with pre–virtual care (13.3% and 7%, respectively) [17], whereas another found a 5% increase in attendance at EPI appointments following virtual care implementation [18]. A more recent study examined the ranges of missed appointments before and after virtual care implementation and found greater variability in the percentage of missed appointments in the post–virtual care (2.7%‐9%) than pre–virtual care implementation (2.8%‐6.4%) [19]. Another study examining EPI services before and after the pandemic noted a significant increase in video appointments offered postpandemic; however, no significant difference in video appointments attended [20]. Given these conflicting findings, more research is needed to evaluate the impact of virtual care implementation on EPI appointment attendance and identify factors that may lead to nonattendance.

We recently completed a study that examined factors associated with attendance at the EPI consultation appointment from 2018 to 2019, when this appointment was offered exclusively in person [5]. We found that older patients and those referred from the ED were less likely to attend the consultation appointment. Additionally, older patients and those who identified as Black or belonging to other racial and ethnic groups were more likely to be referred to EPI services from the ED compared with White patients, indicating that structurally marginalized groups are facing barriers accessing care early in the course of illness (before it becomes an emergency) [5]. While the digital divide and disparities in access to technology may be expected to increase barriers to care [19], it remains unclear if the aforementioned health equity and service use factors continue to affect access to EPI care when services are delivered virtually.

Using retrospective data from a large EPI program, we examined factors associated with attendance at the first EPI consultation appointment following the transition to most of these being scheduled virtually, with a focus on self-reported health equity and service use factors. Initial engagement was defined as attendance at the consultation appointment. We hypothesized that patients who were older, referred from the ED, or identified as Black would be less likely to attend the consultation appointment when it was delivered virtually [5].

## Methods

### Setting

The Centre for Addiction and Mental Health (CAMH) EPI program is the largest in Canada, serving downtown Toronto, a large urban center. The program provides consultation and 3 years of coordinated specialty care for people up to 29 years of age with affective, nonaffective, and substance-induced psychosis. The program receives referrals from the ED, CAMH inpatient units, CAMH outpatient psychiatrists, or externally through primary care providers or external inpatient and outpatient psychiatrists. The ED also houses a “bridging” clinic, where they may triage patients who are determined to be at lower acuity levels during business hours; it is also used to provide short-term follow-up after an inpatient discharge. External referrals are triaged by nurses to appropriate services. The program aims to offer consultation appointments within 2 weeks [21].

### Study Design and Population

This was a retrospective cohort study that followed Strengthening the Reporting of Observational Studies in Epidemiology (STROBE) reporting guidelines for cohort studies [22]. We replicated methods from our previous paper examining initial engagement with in-person services [5]. Electronic medical record (EMR) data were obtained for all patients aged 16 to 29 years who were referred to the CAMH EPI program from April 2020 to December 2020, the period immediately following virtual care implementation. We classified consultation appointments between 2018 and 2019 as “pre–virtual care,” as they were offered exclusively in-person during this period. Appointments between April and December 2020 were classified as “post–virtual care,” as the vast majority were conducted virtually, consistent with institutional practices and jurisdictional restrictions at the time. We focused on this specific period because in-person consultations resumed during subsequent phases of the pandemic. Referral data were excluded from the analysis if the referral was canceled for any reason and for patients who were previously enrolled in the CAMH EPI program. Missing data were manually extracted from EMR charts when possible, and data from second and third referrals were excluded from the analysis. Statistical comparisons were made with 2018 to 2019 data prior to virtual care implementation [5].

### Variables and Missing Data

The primary outcome was rate of attendance at the first consultation appointment. Patients were coded as having not attended if they declined services, could not be reached to schedule their appointment, or did not attend their scheduled appointment. Demographic variables were self-reported by patients through CAMH’s standardized health equity form that is routinely completed around the time of their first appointment and during ED visits and inpatient admissions. These variables included age, gender, racial and ethnic group (“other racial and ethnic groups” included Indigenous, Latin American, Middle Eastern, and other not specified), country of birth, and sexual orientation. Low prevalence categories were combined for data cells of fewer than 5 people to protect the privacy of participants and facilitate statistical comparisons. Recoded variables are outlined in Multimedia Appendix 1. Service use factors were derived from clinical documentation: referral source (inpatient, ED or bridging clinic, and those from outpatient psychiatrists, primary care providers, or other external providers) and days to consult (calculated as the number of days from referral to consultation appointment). We retained “do not know” or “prefer not to answer” responses in the analysis because we considered them valid responses to self-reported health equity questions. Nonresponse data were categorized as missing, and data were missing for three variables (25.9%-27.6%). We performed Little’s MCAR test, which indicated that data were MCAR (*χ*^2^_13_=11.09, *P*=.60). However, we did not impute missing data because it is routinely completed by patients around the time of their first appointment and during ED visits and inpatient admissions. To minimize potential sources of bias, we applied consistent inclusion and exclusion criteria and used standardized EMR data fields to minimize misclassification.

### Statistical Analysis

Descriptive statistics were calculated, including means, SDs, and percentages, to describe the demographic and service use characteristics of the full sample. We used chi-square tests for categorical variables and independent *t* tests for continuous variables to compare service use pre– versus post–virtual care. Effect sizes were calculated using Cohen *d* for continuous variables and Phi (2 × 2 tables) and Cramér *V* (2 × 3 tables) for categorical variables. We used binary logistic regression to model the odds of attendance at the consultation appointment, controlling for demographic (ie, age, gender, racial and ethnic group, sexual orientation, and country of birth) and service use (ie, referral source and days to consult) factors. Simulation work by Vittinghoff and McCulloch (2007) demonstrated that logistic regression can yield reliable estimates with 5 to 9 events-per-variable (EPV) under commonly encountered modeling conditions (ie, when covariates are not extremely sparse, effect sizes are moderate, and covariates are not highly collinear). Our data satisfy all these characteristics, and the candidate covariates were prespecified based on theoretical and clinical consideration [23]. Univariable tests were performed and variables statistically significant at an a priori level of *P*<.20 were included in the multivariable model. Backward stepwise selection was used to determine the final adjusted model by removing variables with a *P*≥.20. This criterion was used as a heuristic to safeguard against type II error during model development, following recommendations suggesting that conservative thresholds (eg, .05 or .10) may prematurely exclude important variables [24]. However, we retained age and gender in the adjusted model, regardless of statistical significance, due to their theoretical and clinical importance. A 2-sided *P*<.05 was considered statistically significant. All statistical analyses were performed using Stata BE (version 17.0; StataCorp) [25].

### Ethical Considerations

This study was approved by the Research Ethics Board at CAMH (#060/2020‐01). Informed consent was not required by CAMH’s Research Ethics Board, as retrospective deidentified EMR data were used for the analysis and posed minimal risk. Data were deidentified and additional efforts were taken to protect the privacy of participants, including combining data cells of fewer than 5 people for low prevalence categories. There was no compensation for participants in this study, as it was an analysis of retrospective EMR data. We have taken efforts to ensure that no identification of individual participants would be possible via the data in the results or supplementary material. There were no images included in the manuscript that could lead to identification of participants.

## Results

### Participant Characteristics

CAMH’s EPI program received 383 patient referrals between April 2020 and December 2020. Referral data that did not meet study eligibility were excluded (70/383, 18.3%), and second and third referrals were filtered out of the analysis (12/313, 3.8%), leaving 301 unique patient referrals. Table 1 displays the demographic and service use characteristics of patients. Patients had a mean age of 23.2 (SD 3.3) years, 71.1% (214/301) identified as male, 29.2% (88/301) identified as White, 40.2% (121/301) identified as heterosexual, and 46.2% (139/301) were born in Canada.

**Table 1.** Characteristics of patients referred to the Centre for Addiction and Mental Health early psychosis intervention program in 2018‐2019 and 2020.

Characteristics	All referrals	
	Pre–virtual care, 2018‐2019 (n=999^a^)	Post–virtual care, 2020 (n=301)
Age (y), mean (SD)	22.5 (3.5)	23.2 (3.3)
Age (y), median (IQR)	22 (20‐25)	23 (21‐26)
Gender, n (%)		
Male	654 (65.5)	214 (71.1)
Female	323 (32.3)	81 (26.9)
Trans, nonbinary, two-spirit, other, or prefer not to answer^b^	22 (2.2)	6 (2.0)
Racial and ethnic group, n (%)		
Asian	199 (19.9)	36 (12.0)
Black	176 (17.6)	55 (18.3)
White	384 (38.4)	88 (29.2)
Other racial and ethnic groups^c^	143 (14.3)	28 (9.3)
Do not know or prefer not to answer	24 (2.4)	16 (5.3)
Missing	73 (7.3)	78 (25.9)
Sexual orientation, n (%)		
Heterosexual	667 (66.8)	121 (40.2)
LGBTQ2S+^d^^,e^	159 (15.9)	41 (13.6)
Do not know or prefer not to answer^e^	54 (5.4)	56 (18.6)
Missing	119 (11.9)	83 (27.6)
Born in Canada, n (%)		
No	294 (29.4)	69 (22.9)
Yes	606 (60.7)	139 (46.2)
Do not know or prefer not to answer	26 (2.6)	15 (5.0)
Missing	73 (7.3)	78 (25.9)
Referral source, n (%)		
Outpatient psychiatrists, PCPs^f^, or other external providers	525 (52.6)	122 (40.5)
ED^g^/Bridging	217 (21.7)	65 (21.6)
Inpatient	257 (25.7)	114 (37.9)
Days to consult, mean (SD)^h^	18.3 (13.9)	12.6 (8.9)
Days to consult, median (IQR)	15 (10-22)	11 (7-15)
Monthly referrals, mean (SD)	44.2 (9.9)	34 (4.4)

a Pre–virtual care data from Polillo et al [5].

b 2018‐2019 data includes two-spirit and prefer not to answer.

c Other racial and ethnic groups include Indigenous, Latin American, Middle Eastern, and other not specified.

d LGBTQ2S+: lesbian, gay, bisexual, trans, queer (or sometimes questioning), and two-spirited.

e 2018‐2019 data includes two-spirit and 2020 data includes prefer not to answer.

f PCP: primary care provider.

g ED: emergency department.

h Includes data only for participants who booked a consultation appointment (n=280).

### Service Use

Service use characteristics are displayed in Tables1  2. Approximately one-fifth of patients (65/301, 21.6%) were referred from the ED or bridging clinic, 37.9% (114/301) from inpatient units, and 40.5% (122/301) from other referral sources. There were significantly higher rates of inpatient referral (114/301, 37.9%) and lower rates of referral from outpatient and other providers (122/301, 40.5%) post–virtual care compared with pre–virtual care (*χ*^2^_2_=18.7, *P*<.001), with a small effect size and moderately narrow CI (Cramér *V*=0.120, 95% CI 0.06 to 0.17). The mean number of days from referral to consultation appointment was 12.6 (SD 8.9), which was significantly lower (*t*_1149_=6.44, 95*%* CI 3.95 to 7.41; *P*<.001) than wait times pre–virtual care (mean 18.3, SD 13.9), with a moderate effect size and narrow CI (Cohen *d*=0.44, 95*%* CI 0.31 to 0.58). The mean number of monthly referrals post–virtual care (mean 34, SD 4.4) was significantly lower (*t*_1298_=−17.3, 95% CI −10.9 to −8.75; *P*<.001) than pre–virtual care (mean 44.2, SD 9.9), with a large effect size and a narrow CI (Cohen *d*=−1.14, 95*%* CI −1.27 to −1.00). Overall, 84.1% (253/301) of patients attended their consultation appointment post–virtual care, which was significantly higher than attendance pre–virtual care (770/999, 77.1%); however, it was a small effect with a moderately wide CI (*χ*^2^_1_=6.71, *φ*=0.072, 95% CI 0.02 to 0.12; *P*=.01). Post–virtual care, patients referred from the ED or bridging clinic had the highest rate of nonattendance (18/65, 27.7%) at the consultation appointment compared with those referred from inpatient units (19/114, 16.7%) or other providers (11/122, 9%).

**Table 2.** Rates of attendance at early psychosis intervention consultation appointments at the Centre for Addiction and Mental Health by referral source in 2018‐2019 and 2020.

Referral source	Outcome of referral					
	Pre–virtual care, 2018-2019^a^			Post–virtual care, 2020		
	Patients, n	Attended consult, n (%)	Did not attend consult^a^, n (%)	Patients, n	Attended consult, n (%)	Did not attend consult^b^, n (%)
All referral sources	999	770 (77.1)	229 (22.9)	301	253 (84.1)	48 (15.9)
Inpatient	257	215 (83.7)	42 (16.3)	114	95 (83.3)	19 (16.7)
ED^c^/Bridging	217	145 (66.8)	72 (33.2)	65	47 (72.3)	18 (27.7)
Outpatient psychiatrists, PCPs^d^, or other external providers	525	410 (78.1)	115 (21.9)	122	111 (91.0)	11 (9.0)

a Pre–virtual care data from Polillo et al [5].

b Nonattendance at consult includes those who declined services, could not be reached for booking, or booked and did not attend.

c ED: emergency department.

d PCP: primary care provider.

### Factors Associated With Attendance at Consultation Appointment

Following univariable tests and stepwise backward selection, identifying as Black (odds ratio 0.45, 95*%* CI 0.21 to 0.97) and being referred from the ED or bridging clinic (odds ratio 0.24, 95*%* CI 0.08 to 0.72) were associated with decreased odds of attendance at the consultation appointment in the final adjusted model (Table 3).

**Table 3.** Logistic regression analysis of factors associated with attendance at early psychosis intervention consultation appointment at the Centre for Addiction and Mental Health during virtual care delivery in 2020 (n=301).

Variable	Attendance at EPI^a^ consultation appointment			
	Univariable		Multivariable	
	OR^b^ (95% CI)	*P* value	OR (95% CI)	*P* value
Age (y)	0.94 (0.85‐1.03)	.18	0.91 (0.80‐1.02)	.11
Gender				
Male	Reference	—^c^	Reference	—
Female, trans, nonbinary, or other	0.98 (0.50‐1.94)	.96	1.24 (0.51‐3.00)	.64
Racial and ethnic group				
Asian^d^	0.65 (0.24‐1.82)	.42	—	—
Black	0.42 (0.18‐0.99)	.046	0.45 (0.21‐0.97)	.04
White	Reference	—	Reference	—
Other racial and ethnic groups, do not know, or prefer not to answer^d^^,^^e^	1.58 (0.48‐5.21)	.45	—	—
Sexual orientation				
Heterosexual	Reference	—	Reference	—
LGBTQ2S+^f^, do not know, or prefer not to answer	1.39 (0.67‐2.88)	.37	—	—
Born in Canada				
Yes	Reference	—	Reference	—
No, do not know, or prefer not to answer	0.91 (0.45‐1.87)	.80	—	—
Referral source				
Outpatient psychiatrists, PCPs^g^, or other external providers	Reference	—	Reference	—
ED^h^/Bridging	0.26 (0.11‐0.59)	.001	0.24 (0.08‐0.72)	.01
Inpatient	0.50 (0.22‐1.09)	.08	0.48 (0.17‐1.41)	.18
Days to consult	1.04 (0.98‐1.10)	.22	—	—

a EPI: early psychosis intervention.

b OR: odds ratio

c Not applicable

d Variables removed from the adjusted model through backward stepwise selection.

e Other racial and ethnic groups include Indigenous, Latin American, Middle Eastern, and other not specified.

f LGBTQ2S+: lesbian, gay, bisexual, trans, queer (or sometimes questioning), and two-spirited.

g PCP: primary care provider

h ED: emergency department

## Discussion

### Principal Findings

Using retrospective cohort data from 301 patients, we found that patients referred to EPI services after the transition to virtual care had higher rates of attendance at the consultation appointment and encountered shorter wait times compared to those referred pre–virtual care. We also found higher rates of inpatient referral and lower rates of referral from outpatient and other providers post–virtual care compared to pre–virtual care. Approximately one-third of patients did not attend their consultation appointment; ED or bridging clinic referrals had the highest rate of nonattendance at the consultation appointment compared with other referral sources. Equity-related and service use factors, notably identifying as Black and being referred from the ED or bridging clinic, were associated with decreased odds of attending the consultation appointment when it was mostly delivered virtually. These findings suggest that virtual care can improve initial engagement in EPI services; however, patients from structurally marginalized groups and those referred from acute sources still face barriers to care, even when appointments are delivered virtually.

### Comparison With Prior Work

We observed a 7% increase in attendance at the EPI consultation appointment following the implementation of virtual care, which is higher than the 5% increase found across all appointments in a prior study [18]. This difference is likely attributable to the focus on the consultation appointment (rather than all appointments). Findings from both studies, when taken together, lend support to virtual care as a tool for improving initial engagement in EPI services, as well as throughout treatment; however, it is important to acknowledge that other factors, such as the COVID-19 lockdown which restricted nonessential outings and may have made youth more available to attend their appointments, may have influenced appointment attendance. We also observed a decrease in wait times from EPI referral to the consultation appointment post–virtual care compared to pre–virtual care, consistent with emerging evidence that virtual care may allow for increased efficiency and streamlined processes [17]. Although the association of shorter wait times with virtual care is an important finding, it is also important to note that monthly referrals to EPI were significantly lower post–virtual care compared with pre–virtual care, which, in turn, could be influencing the shorter wait times. Nevertheless, longer wait times for EPI services have been associated with worse patient outcomes, not only during the initial period of untreated psychosis but also later in treatment [26].

Our results also align with existing research on disparities in virtual care access. Patients referred from the ED or bridging clinic had decreased odds of attending the consultation appointment post–virtual care, consistent with our prior work showing lower attendance rates at in-person initial appointments for those referred from the ED or bridging clinic [5]. Individuals seeking care from acute services may experience greater symptom burden, such as disorganization, paranoia, and a fear of surveillance, which may affect the utilization of virtual care. For example, patients may be too disorganized to navigate connecting online at the prescribed time [27]. Additionally, these patients may be more likely to experience socioeconomic disadvantage, which is linked to increased ED usage, decreased engagement with outpatient mental health care [28], and digital equity barriers. In contrast to findings from our previous study examining in-person attendance [5], we also found that Black patients had a decreased odds of attending the consultation appointment post–virtual care, suggesting that virtual care may amplify health inequities through the digital divide. This is consistent with other research reporting that Black patients were less likely to use telehealth services compared to White patients [29,30], likely due to systemic barriers that can limit access to devices, the internet, or digital literacy skills [31,32]. This is layered upon a long history of racism, oppression, and stigmatization of Black people by the medical system, and ongoing inequities, including a greater risk of police involvement and involuntary hospitalization for Black youth experiencing psychosis [33]. Overcoming systemic barriers to psychosis care for Black patients will require systemic solutions, such as increasing the racial and ethnic diversity of clinicians, ensuring a trauma-informed and anti-racist approach to services, and including lived experience perspectives of Black patients and their families in EPI evaluation [34].

### Implications

These findings have several implications for the design and delivery of EPI services in a postpandemic context. The use of a hybrid model in EPI, integrating virtual care with in-person appointments, can be a way to engage patients in treatment in a way that meets their needs while still maintaining the benefits of an in-person therapeutic relationship. Global surveys of clinicians and clinical leaders show strong support for hybrid models, though challenges exist around infrastructure, training, and digital literacy [35]. Mental health care organizations, including EPI programs, should consider building up technological infrastructure and capacity to support clinicians’ ability to offer hybrid models of care, although we acknowledge that this may be challenging for more low-resourced settings. Additionally, our results showed a shift in referral patterns post–virtual care, with increased inpatient and decreased outpatient referrals. This may reflect delays in early detection of psychosis due to school closures or reduced access to primary care and other outpatient providers. It is clear that outpatient providers represent irreplaceable sources for early detection and EPI access. Leveraging hybrid care models can improve connection to outpatient providers, though consideration of systemic solutions to reduce inequities in digital access is required.

### Limitations

This study has several limitations. Due to the wide CIs in the final adjusted model, the findings should be interpreted with caution. Variables, including race/ethnicity, sexual orientation, and country of birth, had more than 10% of missingness, which was not imputed due to it being self-reported health equity data. There were increases in the percentage of missing data for certain demographic categories from pre–virtual care to post–virtual care, including racial and ethnic group and sexual orientation. These changes highlight potential challenges with the reliability of self-reported data, particularly in the virtual care context. While the overall missingness is reported as more than 10%, the variability in the self-reported health equity data between the two timepoints may affect the accuracy and generalizability of the findings. Future research should explore methods to improve the completeness and reliability of self-reported data within virtual settings. The time period of the study was also a limitation, as it was during the COVID-19 pandemic and may not be representative of virtual care in real-world practice. Thus, findings may be partially related to pandemic effects rather than the transition to virtual care. The use of data from 2020 may also limit the relevance of our findings, given changes in healthcare delivery and policy over time [36]. We were not able to include other relevant patient- and system-level factors, such as socioeconomic status and illness severity, as covariates in the model. These factors may better explain the associations observed in our final adjusted model. For example, lower referral volumes and treatment delays may have led to patients presenting to care when they were more ill and had a higher symptom burden [37]. As such, not having specific data on illness severity limits our ability to understand its potential impact on EPI attendance and engagement and should be an area for future investigation. It is also important to note that patients referred from the ED may differ from those referred through outpatient and inpatient settings [5], limiting the generalizability of our findings across referral sources. Another limitation is that the study setting was a well-resourced hospital with infrastructure and technological support dedicated to virtual care implementation, the findings may not be generalizable to lower-resourced mental health settings.

### Conclusions

This retrospective cohort study of patients referred to EPI services is innovative in that it examines the self-reported health equity and service use factors that may contribute to nonattendance when most EPI appointments are delivered virtually. This is unlike previous studies, which solely focused on differences in attendance rates. Although the time period of the study is a limitation, as it was during the COVID-19 pandemic and may not be representative of virtual care in real-world practice, we found improved attendance rates at the first EPI consultation appointment and shorter wait times, suggesting that virtual care may improve initial engagement in EPI services. Despite this, barriers to care still exist for Black patients and those referred from the ED. The use of a hybrid model can be a way to improve connection to EPI, though targeted approaches are needed to improve the digital divide and ensure that structurally marginalized and high-acuity patients have equitable access to EPI care. Future research should examine virtual engagement in EPI services in a postpandemic context.

## Supplementary material

10.2196/81313 Multimedia Appendix 1 Recoded variables.

10.2196/81313 Checklist 1 STROBE checklist.

## Abbreviations

CAMH: Centre for Addiction and Mental Health
ED: emergency department
EMR: electronic medical record
EPI: early psychosis intervention
STROBE: Strengthening the Reporting of Observational Studies in Epidemiology
