# Supplementary material for: Virtual Delivery of Early Psychosis Care: Retrospective Cohort Study of Factors Associated With Initial Engagement
Source: J Med Internet Res. 2026 Feb 19;28:e81313. doi: 10.2196/81313 (PMC12919743; doi:10.2196/81313)
Supplement: Multimedia Appendix 1 [file jmir-v28-e81313-s001.docx]

**Supplement 1. Recoded Variables**

| Variable | Recoding |
| --- | --- |
| Gender | Male vs. female, trans, non-binary, or other |
| Racial/ethnic group | Asian vs. Black vs. White vs. other racial/ethnic groups, do not know, or prefer not to answer |
| Sexual orientation | Heterosexual vs. LGBTQ2S+, do not know, or prefer not to answer |
| Country of birth | Yes vs. no, do not know or prefer not to answer |
| Referral source | Inpatient vs. ED/bridging vs. other (outpatient psychiatrists, PCPs, or other external providers) |
| Attendance at consultation appointment | Attended vs. did not attend |

Abbreviations: LGBTQ2S+, lesbian, gay, bisexual, trans, queer (or sometimes questioning), and two-spirited; ED, emergency department; PCP, primary care provider.
